# The Efficacy of Coil Embolization to Obtain Intrahepatic Redistribution in Radioembolization: Qualitative and Quantitative Analyses

**DOI:** 10.1007/s00270-019-02351-1

**Published:** 2019-10-24

**Authors:** Ahmed A. Alsultan, Caren van Roekel, Maarten W. Barentsz, Arthur J. A. T. Braat, Pieter Jan van Doormaal, Marnix G. E. H. Lam, Maarten L. J. Smits

**Affiliations:** 1 Department of Radiology and Nuclear Medicine, University Medical Center Utrecht, Utrecht, The Netherlands; 2 Department of Radiology, Erasmus University Medical Center, Rotterdam, The Netherlands

**Keywords:** Transarterial radioembolization, Intrahepatic redistribution, Quantitative analysis

## Abstract

**Purpose:**

To evaluate the efficacy of coil embolization to obtain intrahepatic redistribution in patients undergoing radioembolization.

**Materials and Method:**

All patients treated with radioembolization at our institute were retrospectively analyzed, and all cases in which a tumor-feeding vessel was coil-embolized were selected. Two nuclear medicine physicians visually assessed the effect of redistribution. Furthermore, the redistribution of microspheres was measured by quantifying the activity distributed to the coil-embolized (dependent) segment relative to the other (non-dependent) segments and to the tumor(s) in that segment. Quantitative analysis was performed on post-treatment ^90^Y-PET and ^166^Ho-SPECT using Simplicit^90^Y software. Lesion response was measured according to RECIST 1.1 criteria at 3 months post-treatment.

**Results:**

Out of 37 cases, 32 were suitable for quantitative analysis and 37 for qualitative analysis. In the qualitative analysis, redistribution was deemed successful in 69% of cases. The quantitative analysis showed that the median ratio of the activity to the dependent embolized segments and the non-dependent segments was 0.88 (range 0.26–2.05) and 0.80 (range 0.19–1.62) for tumors in dependent segments compared with tumors in non-dependent segments. Using a cutoff ratio of 0.7 (30% lower activity concentration in comparison with the rest of the liver), 57% of cases were successful. At 3 months post-treatment, 6% of dependent tumors had partial response, 20% progressive disease, and 74% stable disease. In non-dependent tumors, this was, respectively, 16%, 20%, and 64%.

**Conclusion:**

Coil embolization of hepatic arteries to induce redistribution of microspheres has a limited success rate. Qualitative assessment tends to overrate redistribution.

## Introduction

Radioembolization is increasingly used for the treatment of primary and secondary liver tumors. The treatment consists of an intra-arterial injection of microspheres loaded with yttrium-90 (^90^Y) or holmium-166 (^166^Ho). The microspheres are commonly injected in a lobar or segmental fashion [1]. Injection can be challenged by the presence of early bifurcations, replaced or accessory hepatic arteries, and ‘parasitized’ arteries (i.e., non-hepatic arteries contributing to the vascular supply of the liver tumors), or by the proximity to non-target vessels. Therefore, multiple injection positions may be required.

Each injection position requires a change of the vial, microcatheter, and tubing, and the injected activity needs to be adjusted to the target volume. Consequently, radioembolization procedures requiring multiple injection positions are more prone to catheter-related complications and dosing errors. Multiple injection positions are also costly due to the higher material costs and prolonged procedure time.

**Fig. 1:**
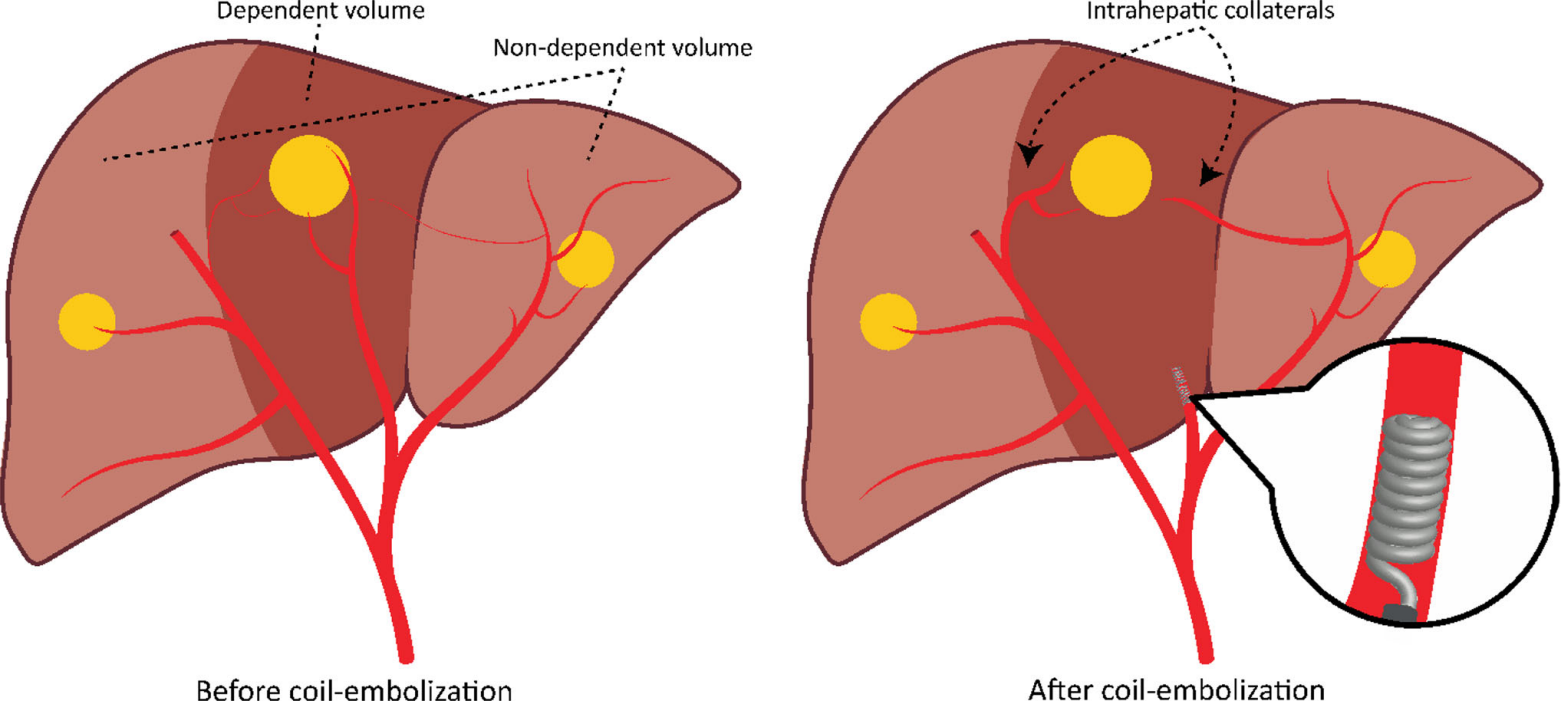
Principle of redistribution. A typical situation with a middle hepatic artery (or segment IV artery) that would require three separate injections in case of whole-liver treatment (right hepatic artery, middle hepatic artery, and left hepatic artery). Coil embolization of the middle hepatic artery can be performed to reduce the number of injection positions and rely on redistribution of microspheres through intrahepatic collaterals

**Fig. 2:**
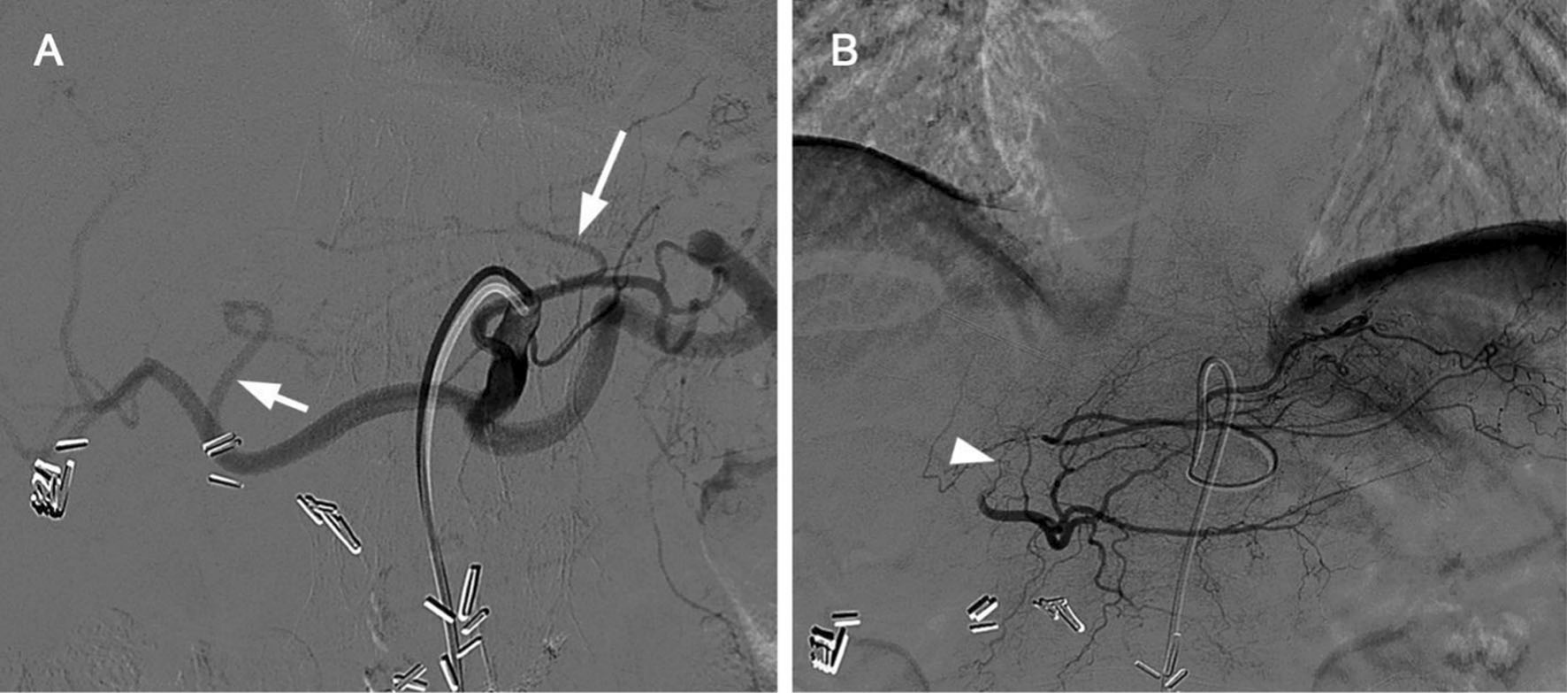
Intrahepatic collateral pathways on DSA. **A** Celiac trunk overview shows the native left hepatic artery (short arrow) and accessory left hepatic artery arising from the left gastric artery (long arrow). **B** Selective angiography from the accessory left hepatic artery shows filling of the native left hepatic artery, demonstrating a patent connection (arrowhead) even without coil embolization

To overcome these problems, techniques are used to reduce the number of injection positions. One of these techniques is embolizing one of the tumor-feeding arteries, leading to redistribution of blood flow through collateral pathways from adjacent hepatic arteries (Figs. 1, 2) [2, 3]. There are three types of redistribution: (1) occlusion of a segmental/subsegmental tumoral feeding artery, (2) occlusion of an aberrant/replaced segmental/lobar artery, and (3) occlusion of a parasitized artery. Various publications have reported on the success of redistribution in radioembolization [4–8].

The aims of this study were to evaluate and quantify the effect of coil embolization of tumor-feeding vessels on the redistribution of blood flow, to assess tumor response, and to study patient and treatment factors that affect redistribution.

## Methods

### Patient Selection and Data Collection

All patients scheduled to undergo radioembolization at our institute for primary or metastatic hepatic cancer between June 2011 and October 2017 were evaluated for inclusion. Radioembolization treatments were performed with both glass (Therasphere^®^, Biocompatibles UK Ltd.) and resin ^90^Y microspheres (SIR-Spheres^®^, Sirtex medical Ltd.), as well as ^166^Ho microspheres (QuiremSpheres^®^, Quirem Medical B.V.). Patients were included if they had undergone embolization of at least one tumor-feeding arterial branch. Patients were excluded when post-treatment imaging was not available.

Both angiography images and cone-beam CT images, acquired during the work-up procedure of the included patients, were reviewed to identify the coil-embolized artery and the liver volume that was vascularized by it (i.e., dependent liver volume). Baseline characteristics were obtained, including type of primary tumor, gender, age, injection sites, and interval between coil embolization and radioembolization.

Our institute’s medical ethics committee waived the need for informed consent for this retrospective study.

### Qualitative Analysis

The distribution of microspheres on post-treatment imaging was analyzed qualitatively by visual assessment performed by two nuclear medicine physicians with > 5 years of experience with radioembolization. Data on gender, age, embolized artery, dependent embolized segment, intended target volume, and relevant information regarding patients’ medical history (e.g., history of hepatic surgery, radio frequency ablation) were provided. Relevant digital subtraction angiography images and cone-beam CT images were also available. All other data were blinded. The redistribution was visually rated on a nominal scale: (1) no redistribution, (2) dubious redistribution, and (3) successful redistribution. Rating was performed independently, and any disagreement was resolved by consensus.

### Quantitative Analysis

The distribution of microspheres was also analyzed quantitatively by measuring the average activity concentration in the dependent segments (i.e., segments that rely on a coil-embolized artery for blood supply), using post-treatment imaging. These activity concentrations were compared to the activity concentrations measured in non-dependent segments (i.e., all liver segments that did not rely on the coil-embolized artery). The activity concentration in dependent tumors was also compared to the activity concentration in non-dependent tumors.

Quantitative analysis was performed using Simplicit^90^Y (Mirada Medical Ltd, Oxford, UK) software. Activity calculations were performed using volumes drawn on contrast-enhanced CT (CECT) images that were registered to low-dose CT images of nuclear imaging datasets. Only rigid transformations were used in the image registration process, i.e., only rotation and translation of the images were performed, preserving the shape and size of the liver.

Volumes of interest (VOI) were manually delineated using the axial reconstruction of a portal venous phase CECT. VOIs of the perfused volume of all injection locations, all measurable tumors (defined as having a diameter ≥ 20 mm), and the dependent segment were drawn. The dependent segment VOIs were preferably drawn using cone-beam CT imaging, otherwise segmentation was performed based on the Couinaud classification of segmental hepatic anatomy. The non-dependent segment VOI was created by subtracting the dependent segment from the whole-liver VOI. The activity concentrations were calculated using the net administered activity (i.e., corrected for residual activity). Activity concentrations in patients treated with holmium were also calculated using Simplicit^90^Y software. As a part of this study, activity measurements obtained in Simplicit^90^Y were compared with measurements made using in-house developed dosimetry software in order to validate the use of Simplicit^90^Y for ^166^Ho-microspheres [9]. The differences were found to be negligible.

Patients in whom not all above-mentioned VOIs could be delineated were excluded from this analysis, as well as cases where accurate registration of CECT to post-treatment imaging was impossible.

### Sequential Lobar Therapy Cases

Patients receiving sequential lobar therapy underwent post-treatment imaging twice (i.e., once for every radioembolization procedure) but were counted as one case. In the visual analysis, both post-treatment scans (i.e., the left and right hemi-liver scans) were assessed separately and the results were subsequently merged, counting the highest score. In the quantitative analysis, the activity concentrations of all VOIs were calculated on both scans and the results were averaged.

### Time Interval

To investigate the effects of the time interval between coil embolization and administration of the microspheres on redistribution, the patients were dichotomized using a threshold of 24 h. This threshold was chosen as almost half of the patients included in this study received treatment within the same day of coil embolization. Segment activity ratios and tumor activity ratios were then compared. Patients receiving sequential lobar treatment were excluded from this subgroup analysis.

### Response Evaluation

Anatomic tumor response was assessed on CECT as per RECIST 1.1. The longest tumor diameter (LTD) was measured in lesions > 1 cm. LTD measurements of all dependent lesions as well as the two largest non-dependent lesions were recorded at baseline and follow-up. Complete response (CR) was defined as an LTD reduction of 100%, partial response (PR) as a reduction of < 100% and ≥ 30%, progressive disease (PD) as an increase ≥ 20% and an absolute increase of ≥ 5 mm, and stable disease (SD) when the LTD change would not qualify for PD nor PR.

### Statistical Analysis

Descriptive statistics were used as proportions and medians with ranges. Ratios of activity concentrations were calculated between dependent and non-dependent segments (the segment ratio) and dependent and non-dependent tumors (the tumor ratio). Since there is no definition of successful redistribution, the success rates for a 10%, 20%, and 30% difference in activity concentration between the dependent and non-dependent volumes were calculated, corresponding to dose ratios of 0.9, 0.8, and 0.7, respectively. Ratios in the time interval analysis were compared with an independent samples *t* test. Inter-rater reliability was evaluated by means of a weighted Cohen’s kappa. All statistical analyses were performed with IBM SPSS Statistics for Windows, version 22 (IBM Corp., Armonk, N.Y., USA).

## Results

**Fig. 3:**
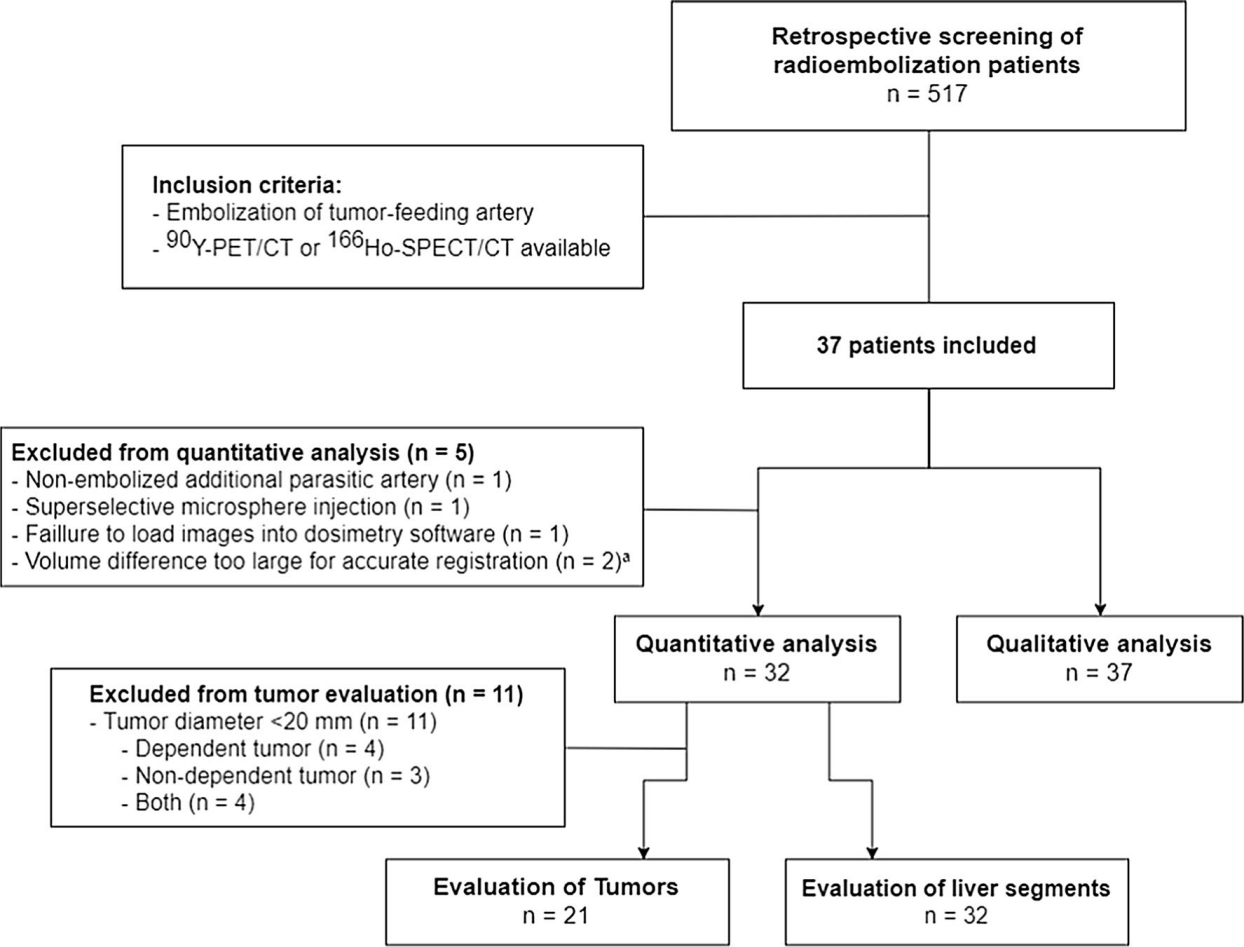
Flow chart of study patients. ^a^Significant hypertrophy of the contralateral lobe occurred in patients that were treated sequentially, making rigid registration with pre-treatment CT imaging impossible

Within the studied timeframe, a total of 517 radioembolization procedures were performed at our institute, of which 37 patients were selected for this study (Fig. 3). In most cases (*n* = 36), microcoils (Interlock™ coils and ‘Figure 8’ coils, Boston Scientific) were used as embolization agent, and in one case, cyanoacryl glue (Histoacryl^®^, B. Braun Surgical S.A.) was used. The baseline characteristics are shown in Table 1. Cone-beam CT images were available in 27 of 37 cases. In 10 cases, cone-beam CT series were acquired after selective injection of contrast agent in the artery that was to be coiled and were helpful in delineating the dependent volume.

**Table 1:** Baseline characteristics

Baseline/treatment characteristics	Value
Mean age in years ± SD	61 ± 9
Gender	
Male	20 (54%)
Female	17 (46%)
Primary neoplasm	
Colorectal carcinoma	17 (46%)
Neuroendocrine tumor	11 (30%)
Cholangiocarcinoma	3 (8%)
Breast carcinoma	2 (5%)
Hepatocellular carcinoma	1 (3%)
Other	3 (8%)
Embolized artery	
MHA/segment IV artery	23 (62%)
aLHA	9 (24%)
rLHA	1 (3%)
Parasitized artery^a^	4 (11%)
Embolization method	
Microcoil^b^	36 (97%)
Cyanoacryl glue	1 (3%)
Segments involved per case	
IV	25 (68%)
II	4 (11%)
II and III	2 (5%)
II, III and IV	2 (5%)
I	1 (3%)
I and VIII	1 (3%)
II and IV	1 (3%)
VII	1 (3%)
Type of microsphere	
Yttrium-90	21 (57%)
Resin	14 (38%)
Glass	7 (19%)
Holmium-166	16 (43%)
Treatment	
Whole liver	23 (62%)
Sequential lobar^c^	5 (14%)
Right lobe only^d^	6 (16%)
Left lobe only^e^	3 (8%)

Age displayed in mean with standard deviation

*MHA* middle hepatic artery, *aLHA* accessory left hepatic artery, *rLHA* replaced left hepatic artery

^a^Right inferior phrenic artery (*n* = 3), right internal mammary artery (*n* = 1)

^b^Interlock™ detachable embolization coils and ‘Figure 8’ pushable coils (Boston Scientific, Marlborough, USA)

^c^Median interval between sequential treatments was 53.5 days

^d^After right hemi-hepatectomy (*n* = 1)

^e^After left hemi-hepatectomy (*n* = 1)

### Qualitative Analysis

Redistribution was rated as successful in 26/37 (70%) cases and dubious in 5/37 (14%) cases, and no redistribution was found in 6/37 (16%) cases. Inter-rater agreement was considered high (*κ* = 0.82).

### Quantitative Analyses

**Fig. 4:**
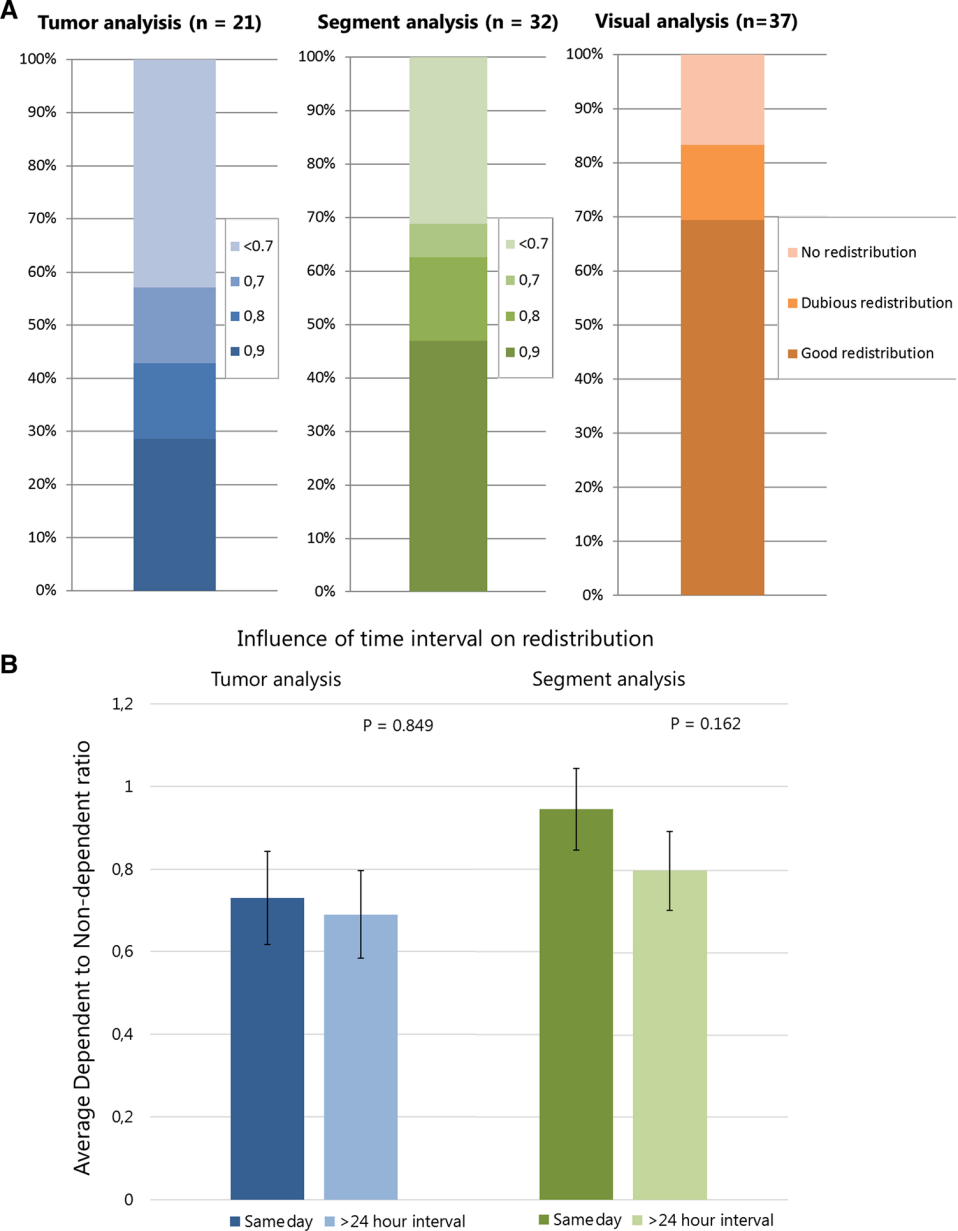
**A** Visual representation of the proportion of successful redistribution cases in both the quantitative and the visual analysis. In the quantitative analysis, success rate was determined based on cutoff values representing activity concentration differences of 10%, 20%, and 30%. **B** Bar chart of the averages in tumor and segment activity ratios for patients treated on the same day after coil embolization versus patients treated after a > 24-h interval

Five patients were excluded from the quantitative analyses. One patient had an additional parasitized artery that could not be coil-embolized, one had a superselective injection of microspheres, in which the healthy liver VOI could not be determined, one had corrupted post-treatment imaging files, and two were treated sequentially and had a large volume increase in one liver lobe making accurate image co-registration impossible. The median ratio of the dependent to non-dependent segment activity concentration was 0.88 (range 0.26–2.05). This means that the activity concentration in the coiled segments amounted to 88% of the activity concentration to the rest of the treated volume. For tumors, the median ratio was 0.80 (range 0.19–1.62). Success rates for redistribution based on activity concentration (using cutoff ratios of 0.9, 0.8, and 0.7) were 29%, 43%, and 57%, respectively (Table 2; Fig. 4A).

**Table 2:** Quantitative analysis

	Activity ratios		Success rate of redistribution					
			Tumor			Segment		
	Tumor	Segment	0.9 cutoff	0.8 cutoff	0.7 cutoff	0.9 cutoff	0.8 cutoff	0.7 cutoff
All patients	0.80 (0.19–1.62)	0.88 (0.26–2.05)	6/21 (29%)	9/21 (43%)	12/21 (57%)	15/32 (47%)	20/32 (63%)	22/32 (69%)
Embolized artery								
MHA/segment IV artery	0.80 (0.19–1.62)	0.89 (0.42–2.05)	4/13 (31%)	6/13 (46%)	9/13 (69%)	10/20 (50%)	13/20 (65%)	14/20 (70%)
aLHA/rLHA	0.50 (0.32–1.41)	0.82 (0.37–1.42)	2/5 (40%)	2/5 (40%)	2/5 (40%)	4/8 (50%)	4/8 (50%)	5/8 (64%)
Parasitized artery	0.49 (0.33–0.84)	0.85 (0.26– 0.93)	0/3	1/3 (33%)	1/3 (33%)	1/4 (25%)	3/4 (75%)	3/4 (75%)
Primary neoplasm								
CRC	0.83 (0.40–1.41)	0.97 (0.26–1.47)	3/11 (27%)	6/11 (55%)	8/11 (72%)	7/14 (50%)	10/14 (71%)	11/14 (79%)
NET	0.49 (0.18–0.80)	0.72 (0.53–1.45)	0/5	0/5	0/5	3/9 (33%)	4/9 (44%)	5/9 (56%)

Medians of the activity ratios are displayed with the range between parentheses. The number of successful redistributions was calculated using cutoff values based on activity concentration decreases of 10, 20, and 30%

*MHA* middle hepatic artery, *aLHA* accessory left hepatic artery, *rLHA* replaced left hepatic artery, *CRC* colorectal carcinoma, *NET* neuroendocrine tumor

### Influencing Factors

Redistribution of segment IV arteries showed the highest rate of successful redistribution to the tumors (31%, 46%, and 69%, using cutoff ratios of 0.9, 0.8, and 0.7, respectively) and parasitized arteries the lowest (0%, 33%, and 33%). There was no notable difference in success rates between the microspheres used (^90^Y glass, ^90^Y resin, or ^166^Ho microspheres). Comparison between the two largest tumor categories, colorectal carcinoma (CRC) and neuroendocrine tumor (NET), showed markedly lower success rates in NET patients (27%, 55%, 72% vs 0%, 0%, 0%).

In parasitized arteries, 0/4 were deemed successful on the visual assessment, 1/4 (25%) was dubious and 3/4 (75%) were unsuccessful. In the quantitative analysis the success rate was 0%, 33%, 33%, using cutoff ratios of 0.9, 0.8, and 0.7, respectively.

### Time Interval

A total of 28 patients were included in the time interval subgroup analysis. Fourteen of which had coil embolization performed on the same day as the treatment procedure, while the comparison group had a median time interval of 10 days (2–32 days). Mean activity ratios in patients treated on the same day were higher than those in the comparison group, respectively, 0.94 versus 0.80 in segment ratios and 0.72 versus 0.69 in tumor ratios; however, the differences were not statistically significant (Fig. 4B).

### Response Evaluation

Lesion-based anatomic response assessment was possible in 31/37 patients. Out of 6 excluded patients, two were not physically able to undergo follow-up imaging, two had follow-up scans that were of suboptimal quality, one only had MR imaging performed, and one patient only had lesions smaller than 1 cm. A total of 91 lesions were evaluated. Out of 35 dependent lesions, 2 (6%) had partial response, 7 (20%) progressed, and 26 (74%) were stable, and out of 56 non-dependent lesions, 9 (16%) had partial response, 11 (20%) progressed, and 36 (64%) were stable (Table 3).

**Table 3:** Lesion response on contrast-enhanced CT at 3 months post-treatment

	Complete response	Partial response	Stable disease	Progressive disease
Dependent tumor	0	2/35 (6%)	26/35 (74%)	7/35 (20%)
Non-dependent tumor	0	9/56 (16%)	36/56 (64%)	11/56 (20%)

## Discussion

This study aimed to evaluate the use of coil embolization for inducing redistribution of hepatic blood flow in radioembolization, by qualitatively and quantitatively analyzing the post-treatment distribution of microspheres, as well as comparison of tumor response. Visual assessment of post-treatment imaging found that 70% of redistribution cases had a similar distribution of microspheres in the dependent and non-dependent segments. However, quantitative assessment demonstrated notably lower absorbed doses in both dependent tumors and segments, and 71% of dependent tumors had an activity concentration that was ≥ 10% lower than their non-dependent counterparts. In both groups, an equal percentage of tumors showed progression; however, the dependent tumors had a lower rate of partial response compared to non-dependent tumors, 6% versus 16%, respectively.

Several studies have previously reported on the redistribution method [4–8]. Three studies visually assessed blood flow redistribution. Lauenstein et al. and Spreafico et al. examined the appearance of collaterals on DSA after coil embolization, as well as the visual presence of ^99m^Tc-MAA or ^90^Y-microspheres in the dependent segments [5, 7]. Redistribution of flow was found in 89% (24/27) and in 100% (*n* = 17) of cases, respectively. Bilbao et al. [8] assessed and scored the accumulation of ^99m^Tc-MAA in the dependent tumors. ^99m^Tc-MAA activity was visually present in 95% (23/24) of the dependent tumors. In 66% (16/24) of patients, the distribution of ^99m^Tc-MAA in dependent tumors was considered similar to the non-dependent segments, which was in concordance with the findings of our visual assessment.

Other studies evaluated the efficacy of redistribution by the assessment of the treatment response and found favorable response rates in the dependent tumors [4, 6–8]. Spreafico et al. [7] found an overall response rate of 100% (3 CR, 8 PR, and 6 SD, according to mRECIST) in dependent tumors at 3 months after treatment. Abdelmaksoud et al. [6] compared tumor response in dependent tumors to their non-dependent counterparts and found inferior response in only one case out of twenty-two (4.5%). While it does support the efficacy of radioembolization treatment in tumors with redistributed blood flow, the endpoint of tumor response does not provide insight into the differences in activity distribution.

**Fig. 5:**
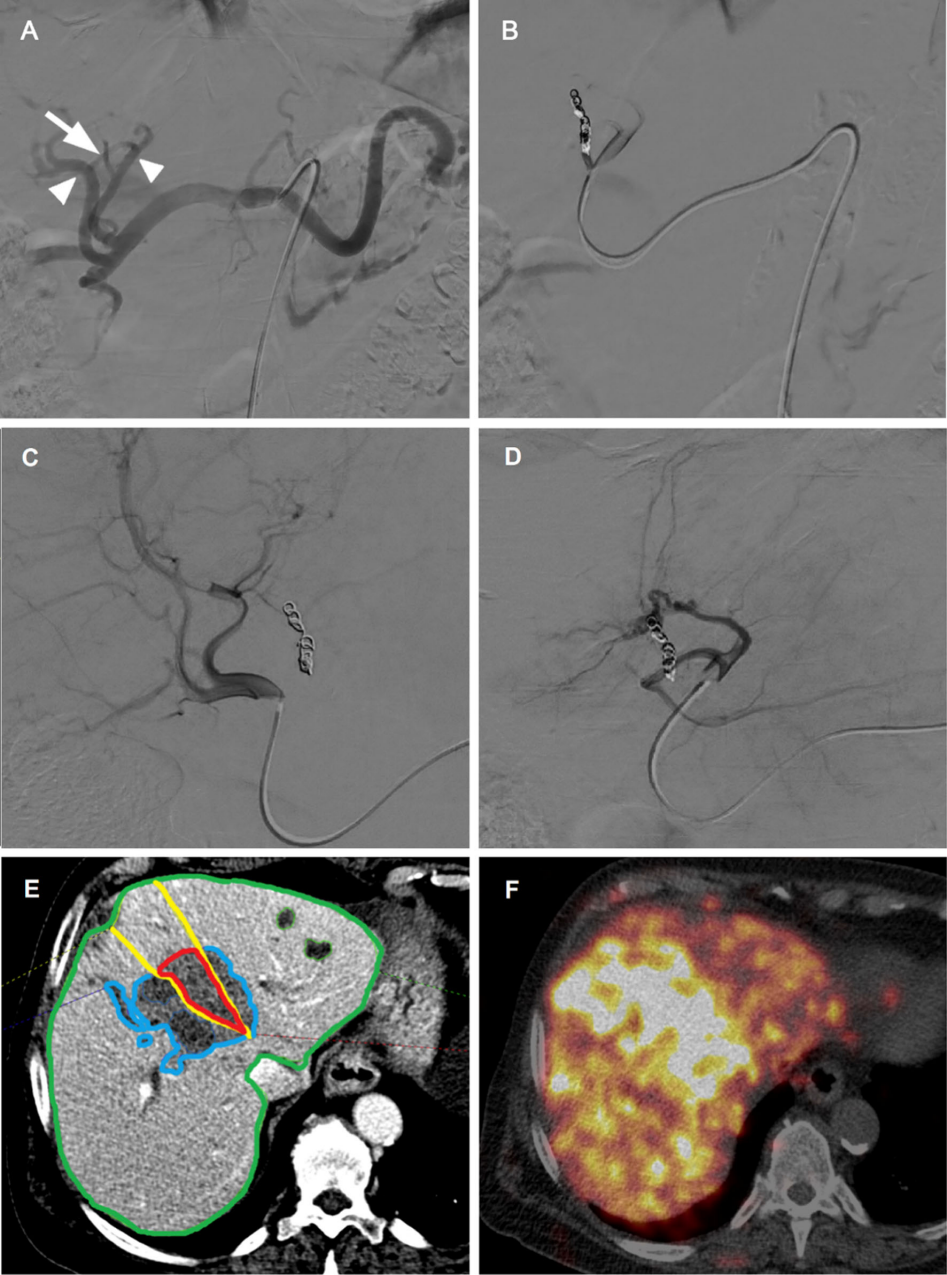
Example of successful redistribution in a patient with cholangiocarcinoma. **A** DSA showing the liver vasculature including the left hepatic artery origin of the segment IV branch (white arrow), as well as the future microsphere injection positions (white arrowheads). **B** Coil embolization of the segment IV branch. **C** Injection position in the RHA post-coil embolization. **D** Injection position in the LHA post-coil embolization. **D** Volumes of interest drawn using Simplicit^90^Y™ software, the dependent segment (IV) was drawn based on Couinaud’s classification of segmental anatomy. **E**^90^Y-PET/CT after treatment demonstrates a high concentration of microspheres throughout the liver, especially in segment IV. *p* = .162

**Fig. 6:**
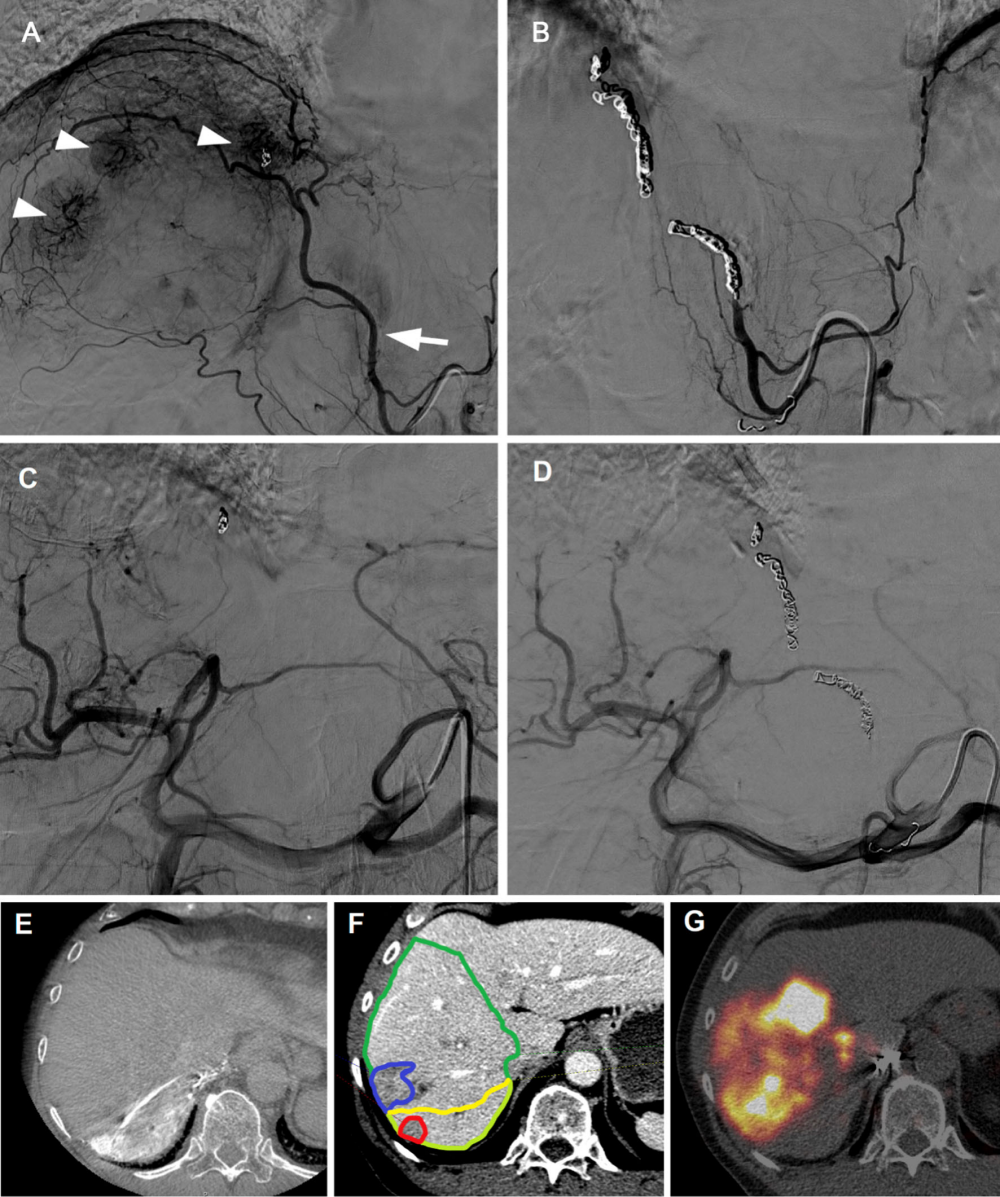
Example of poor redistribution. **A** DSA showing parasitized blood supply to several liver tumors (arrow heads) from the right inferior phrenic artery (arrow). An old microcoil from a prior procedure in another hospital is also visible. **B** Coil embolization of the phrenic artery. **C** Celiac trunk DSA prior to coil embolization of the phrenic artery. **D** Celiac trunk DSA post-coil embolization. **E** Cone-beam CT of the right phrenic artery shows enhancement of tumors in segment VII (prior to coil embolization). **F** Volumes of interest drawn in Simplicit^90^Y software; the liver volume supplied by the phrenic artery segment was delineated using cone-beam CT data. **G**^90^Y-PET/CT after injection of ^90^Y-microspheres in the right hepatic artery shows no redistribution to segment VII

Subgroup analysis showed that middle hepatic artery/segment IV artery redistribution was most successful, which may be attributable to the central location in the liver and the potential intrahepatic collaterals that can reroute blood flow from both the right and the left hepatic artery (Fig. 5). Success rates for obtaining redistribution were lowest when parasitized arteries were embolized (Fig. 6). A possible explanation is that these arteries were (in most cases) newly recruited by the tumorous process and did not (yet) have adequate collateral connections with the adjacent hepatic arteries. In our experience, parasitized arteries require a more distal embolization to prevent the distal segment to recruit blood from other branches (of the parasitized artery). For intrahepatic branches, a proximal embolization suffices in general.

In our comparison between primary tumor types, we found substantially better redistribution rates in CRC compared to NET metastases. This difference was most pronounced in the tumor analysis, in which all NET patients had an absorbed dose difference of ≥ 30%. This was expected to some extent, as hypervascular tumors are more likely to recruit parasitized arteries. Nonetheless, this could not account for the entire difference, as only one NET case involved a parasitized artery. Perhaps also the hypervascular nature of the tumors makes these tumors more prone to under-dosing after redistribution. In addition, the sample size is quite small. Other primary tumor type samples were too small for comparison.

In some of the cases, coil embolization and the injection of microspheres took place on the same day. All other patients had a time interval between coil embolization and treatment of up to 4 weeks. In contrast to what we expected, a longer interval between coil embolization and treatment did not result in a higher success rate. In fact, patients who were embolized and treated on the same day had higher activity ratios, although not statistically significant. These findings suggest that coil embolization to obtain redistribution is even feasible in a 1-day treatment setting. It is important to note that all ‘same day patients’ received treatment with ^166^Ho microspheres. However, this higher success rate was not found when comparing ^166^Ho to ^90^Y microspheres.

What this study adds to the existing literature is the quantitative analysis to evaluate the actual microsphere distribution post-treatment, and the use of ^90^Y-PET/CT instead of ^90^Y-Bremsstrahlung-SPECT as it offers better spatial resolution and contrast for optimized quantification of ^90^Y-activity [10, 11].

The study had several limitations that were mainly related to the quantitative analyses. Quantification was performed using Simplicit90Y software both for yttrium-90 and for holmium-166 patients. While distribution comparison is achievable between these groups, the absolute absorbed dose could not be reported because Simplicit90Y is formally not suitable for holmium-166 dosimetry. Although the results of this study show that the uptake in dependent liver volumes is generally low, this does not necessarily mean that this uptake is below the ‘accepted’ therapeutic ranges. Registration errors occurred when fusing the CECT and the post-treatment images, especially in patients with multiple small bilobar tumors. Furthermore, small errors were introduced due to manual segmentation, and heterogeneity due to the use of multiple microsphere types, and the use of two different post-treatment imaging modalities (i.e., ^90^Y-PET/CT and ^166^Ho-SPECT/CT). Lastly, the study was limited by its retrospective nature as well as small sample size.

Based on the results of this study, we recommend using the redistribution technique only when deemed absolutely necessary, e.g., if otherwise the injection position would be unstable or include non-target vessels. The best results are achieved in coil embolization of the segment IV artery. Coil embolization of parasitized arteries showed the least favorable redistribution of microspheres, and caution is therefore advised when performing redistribution on hypervascular tumors.

In conclusion, visual evaluation of post-treatment imaging tends to overestimate the effect of redistribution. Quantitative analysis demonstrated significantly lower absorbed doses in redistributed dependent parts of the liver. Tumor response was slightly lower in the redistributed tumors.

## Abbreviations

^166^Ho: Holmium-166
^90^Y: Yttrium-90
^99m^Tc: Technetium-99m
aLHA: Accessory left hepatic artery
CECT: Contrast-enhanced CT
CR: Complete response
CRC: Colorectal carcinoma
DSA: Digital subtraction angiography
MHA: Middle hepatic artery
NET: Neuroendocrine tumor
PD: Progressive disease
PR: Partial response
rLHA: Replaced left hepatic artery
SD: Stable disease
VOI: Volume of interest
